# Supplementary material for: Examining the uptake, retention, and effectiveness of a national online type 2 diabetes self-management intervention in England (Healthy Living): A retrospective cohort study
Source: PLoS One. 2026 Jun 3;21(6):e0348266. doi: 10.1371/journal.pone.0348266 (PMC13232854; doi:10.1371/journal.pone.0348266)
Supplement: S1 Table — (PDF) [file pone.0348266.s001.pdf]

Table S1. Baseline characteristics of the study groups

	NDA Controls (Group 1) N=3,707,670	HL account registrants (Group 2) N=27,010	HL account activators (Group 3) N= 21,820	HL attendees (Group 4) N=8,375	HL completers (Group 5) N=990
Proportion of the total English T2DM population (all groups), %	99.3%	0.7%	0.6%	0.2%	0.03%
**Age,** (years) mean (±SD)	64.9 (±13.9)	59.3 (±11.8)	58.8 (±11.8)	58.9 (±11.4)	58.8 (±11.1)
**Sex**, N (%)					
Male	2,068,870 (56%)	11,770 (44%)	9,480 (43%)	3,240 (39%)	385 (39%)
Female	1,638,800 (44%)	15,240 (56%)	12,340 (57%)	5,135 (61%)	605 (61%)
**Ethnicity**, N (%)					
Asian	590,120 (16%)	2,615 (10%)	1,970 (9%)	510 (6%)	40 (4%)
Black	198,980 (5%)	1,225 (4%)	915 (4%)	260 (3%)	20 (2%)
Mixed	45,990 (1%)	320 (1%)	235 (1%)	70 (1%)	5 (0.5%)
Other	62,020 (2%)	285 (1%)	220 (1%)	70 (1%)	5 (0.5%)
White	2,810,570 (76%)	22,570 (84%)	18,485 (85%)	7,470 (89%)	920 (93%)
**Deprivation (IMD)**, N (%)					
IMD Q1 (Most deprived)	893,730 (24%)	4,965 (18%)	3,900 (18%)	1,425 (17%)	170 (17%)
IMD Q2	830,800 (22%)	5,300 (20%)	4,240 (19%)	1,605 (19%)	180 (18%)
IMD Q3	762,805 (21%)	5,505 (20%)	4,450 (20%)	1,725 (21%)	195 (19%)
IMD Q4	669,730 (18%)	6,015 (22%)	4,900 (23%)	1,910 (23%)	210 (21%)
IMD Q5 (Least deprived)	550,610 (15%)	5,235 (19%)	4,335 (20%)	1,715 (20%)	240 (24%)
**Baseline BMI** (kg/m^2)					
Mean (±SD)	31.4 (±6.9)	33.5 (±7.3)	33.7 (±7.4)	33.8 (±7.4)	34.1 (±7.3)
Median (IQR)	30.5 (IQR: 26.6, 35.1)	32.5 (IQR: 28.3, 37.6)	32.6 (IQR: 28.4, 37.8)	32.8 (IQR: 28.6, 37.9)	33.1 (IQR: 29.1, 38.3)
**Smoking status**, N (%)					
Current smoker	520,640 (14%)	2,315 (9%)	1,820 (8%)	720 (9%)	65 (7%)
Ex-smoker	1,292,240 (35%)	9,475 (35%)	7,670 (35%)	3,040 (36%)	355 (36%)
Non-smoker (history unknown)	78,310 (2%)	590 (2%)	485 (2%)	190 (2%)	25 (2%)
Never smoker	1,816,490 (49%)	14,630 (54%)	11,850 (55%)	4,435 (53%)	545 (55%)
**Diabetes duration, years**					
Mean (±SD)	8.1 (±7.9)	6.0 (±6.7)	5.8 (±6.6)	4.9 (±6.2)	4.4 (±6.1)
Median (IQR)	6.0 (IQR: 1.0, 13.0)	4.0 (IQR: 0.0, 10.0)	3.0 (IQR: 0.0, 10.0)	2.0 (IQR: 0.0, 8.0)	1.0 (IQR: 0.0, 7.0)
**Referral Route**, N (%)					
Public beta - Hub referral (NWL)	N/A	390 (1%)	280 (1%)	50 (0.6%)	5 (0.5%)
Self-Referral landing page (Private)	N/A	5,090 (19%)	3,715 (17%)	1,295 (16%)	175 (18%)
Self-Referral landing page (Public)	N/A	21,515 (80%)	17,810 (82%)	7,020 (84%)	810 (82%)
GP Referral	N/A	15 (0.1%)	15 (0.1%)	10 (0.1%)	5 (0.5%)
**Baseline HbA1c** mmol/mol, mean (±SD)	59.5 (±17.6)	62.1 (±18.2)	62.1 (±18.1)	61.9 (±18.1)	62.3 (±19.7)
**Baseline HbA1c** %, mean (±SD)	7.6 (±1.6)	7.8 (±1.7)	7.8 (±1.7)	7.8 (±1.7)	7.9 (±1.8)
**Baseline SBP**, mmHg, mean (±SD)	132.5 (±14.1)	131.4 (±13.5)	131.3 (±13.4)	131.3 (±13.3)	130.8 (±13.3)
**Baseline DBP,** mmHg, mean (±SD)	76.7 (±9.5)	78.4 (±9.1)	78.5 (±9.1)	78.6 (±9.0)	78.4 (±9.0)

	NDA Controls (Group 1) N=3,707,670	HL account registrants (Group 2) N=27,010	HL account activators (Group 3) N= 21,820	HL attendees (Group 4) N=8,375	HL completers (Group 5) N=990
**Baseline total cholesterol,** mmol/L					
Mean (±SD)	4.1 (±1.3)	3.3 (±1.8)	3.2 (±1.8)	3.1 (±1.8)	3.3 (±1.7)
Median (IQR)	4.0 (IQR: 3.3, 4.9)	3.4 (IQR: 1.4, 4.5)	3.31 (IQR: 1.4, 4.5)	3.1 (IQR: 1.4, 4.4)	3.4 (IQR: 1.5, 4.5)
**Baseline serum creatinine,** µmol/L, mean (±SD)	82.7 (±36.6)	75.4 (±28.8)	74.9 (±27.1)	73.4 (±23.9)	72.9 (±23.6)
**Offered DM education programme**					
Yes	2,312,270 (62%)	21,160 (78%)	17,110 (78%)	6,585 (79%)	800 (81%)
Unspecified	1,395,400 (38%)	5,850 (22%)	4,710 (22%)	1,795 (21%)	190 (19%)
**Attended DM education programme**					
Yes	371,375 (10%)	5,270 (19%)	4,305 (20%)	1,530 (18%)	190 (19%)
Unspecified	3,336,300 (90%)	21,740 (81%)	17,515 (80%)	6,845 (82%)	800 (81%)
**Comorbidities**, N (%)					
**Ischaemic heart disease (IHD)**					
Yes	584,235 (16%)	2,460 (9%)	1,860 (8%)	630 (7%)	70 (7%)
Unknown	3,123,440 (84%)	24,550 (91%)	19,965 (92%)	7,745 (93%)	920 (93%)
**History of CVD admission**					
Yes	72,735 (2%)	1,930 (7%)	1,475 (7%)	525 (6%)	55 (6%)
Unknown	3,634,940 (98%)	25,080 (93%)	20,345 (93%)	7,850 (94%)	935 (94%)
**Learning disability (LD)**					
Yes	27,505 (0.7%)	65 (0.2%)	45 (0.2%)	20 (0.2%)	5 (0.5%)
Unknown	3,680,170 (99.3%)	26,945 (99.8%)	21,775 (99.8%)	8,355 (99.8%)	985 (99.5%)
**Severe mental illness (SMI)**					
Bipolar disorder	30,605 (0.8%)	260 (1%)	215 (1%)	55 (0.7%)	10 (1%)
Schizophrenia	47,795 (1%)	135 (0.5%)	100 (0.5%)	25 (0.3%)	5 (0.5%)
Other psychosis	11,205 (0.3%)	50 (0.2%)	45 (0.2%)	15 (0.1%)	5 (0.5%)
SMI Dx not provided	3,618,070 (98%)	26,570 (98%)	21,470 (98%)	8,285 (99%)	975 (99%)
**Baseline medications**, N (%)					
Antihypertensives	2,517,405 (68%)	16,880 (63%)	13,455 (62%)	5,045 (60%)	580 (59%)
Insulin	437,925 (12%)	3,015 (11%)	2,325 (11%)	715 (9%)	90 (9%)
Non-insulin diabetes drugs	2,453,930 (66%)	21,605 (80%)	17,365 (80%)	6,335 (76%)	705 (71%)
Statins	2,541,865 (69%)	18,020 (67%)	14,305 (66%)	5,230 (63%)	595 (60%)

The table presents imputed data (imputation #1).
In accordance with mandatory data provider Statistical Disclosure Control (SDC) rules (such as, rounding and small number suppression), individual categories may not sum to the total, and percentages may not sum to 100%.
BMI: body mass index; CVD: cardiovascular disease; DBP: diastolic blood pressure; HbA1c: glycated haemoglobin; HL: Healthy Living; IHD: ischaemic heart disease; IMD Q: index of multiple deprivation quintile; NDA: National Diabetes audit; SBP: systolic blood pressure; DM: diabetes.
