# Supplementary material for: Examining the uptake, retention, and effectiveness of a national online type 2 diabetes self-management intervention in England (Healthy Living): A retrospective cohort study
Source: PLoS One. 2026 Jun 3;21(6):e0348266. doi: 10.1371/journal.pone.0348266 (PMC13232854; doi:10.1371/journal.pone.0348266)
Supplement: S2 Table — (PDF) [file pone.0348266.s002.pdf]

Table S2. Logistic regression model for 'Attendance' using total time spent on the website (total duration in minutes) measure as binary outcome of total time being ≤ median of 6.85 minutes (reference group) vs. > median time (N= 21,440 HL participants with usage data)

- Total time spent on the website ≤ median time, N= 10,730 (reference group)
- Total time spent on the website > median time, N= 10,710

Variable	Odds Ratio	Std. Err.	t	P>|t|	[95% Conf. Interval]
**Age**	1.011	0.002	6.74	0.000	1.008 – 1.014
**Sex (**Female**)**	1.330	0.042	9.00	0.000	1.250 – 1.415
**Ethnicity** (Vs. White)					
Asian	0.534	0.029	-11.51	0.000	0.480 – 0.594
Black	0.725	0.053	-4.42	0.000	0.629 – 0.837
Mixed	0.772	0.106	-1.89	0.059	0.590 – 1.010
Other	0.925	0.132	-0.55	0.583	0.699 – 1.222
**IMD quintile (**Vs. IMD Q1 Most deprived**)**					
Q2	1.048	0.048	1.03	0.303	0.958 – 1.146
Q3	1.014	0.046	0.31	0.754	0.928 – 1.109
Q4	1.004	0.045	0.10	0.923	0.920 – 1.097
Q5 (Least deprived)	1.045	0.049	0.94	0.347	0.954 – 1.145
**Smoking status** (vs. Never smoker)					
Current smoker	1.045	0.059	0.78	0.437	0.935 – 1.168
Ex-smoker	1.050	0.034	1.50	0.133	0.985 – 1.119
Non-smoker (unknown)	0.995	0.103	-0.05	0.962	0.812 – 1.219
**BMI**, kg/m2	0.996	0.002	-1.64	0.101	0.992 – 1.001
**Diabetes duration,** years	0.972	0.003	-10.99	0.000	0.967 – 0.977
**Baseline HbA1c,** mmol/mol	1.004	0.001	4.94	0.000	1.002 – 1.006
**Baseline total cholesterol,** mmol/L	0.979	0.008	-2.51	0.012	0.962 – 0.995
**Baseline SBP,** mmHg	0.999	0.001	-0.70	0.483	0.996 – 1.002
**Baseline DBP,** mmHg	0.998	0.002	-0.78	0.433	0.994 – 1.002
**Baseline serum creatinine,** μmol/L	1.000	0.001	0.12	0.901	0.999 – 1.001
**Ischaemic heart disease (IHD)**	0.930	0.056	-1.21	0.227	0.827 – 1.046
**History of CVD admission**	0.976	0.064	-0.38	0.707	0.859 – 1.109
**Learning disability (LD)**	0.830	0.257	-0.60	0.546	0.452 – 1.523
**Severe mental illness (SMI)** (vs. SMI not provided)					
Bipolar disorder	0.682	0.099	-2.65	0.008	0.514 – 0.905
Schizophrenia	0.715	0.153	-1.57	0.115	0.470 – 1.086
Other psychosis	0.765	0.246	-0.84	0.404	0.407 – 1.435
**Baseline antihypertensives**	1.014	0.033	0.44	0.659	0.952 – 1.080
**Baseline insulin**	0.853	0.043	-3.17	0.002	0.773 – 0.941
**Baseline non-insulin antidiabetic agents**	0.719	0.027	-8.85	0.000	0.668 – 0.773
**Baseline statins**	0.914	0.031	-2.68	0.007	0.856 – 0.976
**_cons (Constant)**	0.924	0.200	-0.37	0.715	0.605 – 1.412

BMI: body mass index; CVD: cardiovascular disease; DBP: diastolic blood pressure; HbA1c: glycated haemoglobin; HL: Healthy Living; IHD: ischaemic heart disease; IMD Q: index of multiple deprivation quintile; NDA: National Diabetes audit; SBP: systolic blood pressure; DM: diabetes.

Model goodness of fit measured on imputation *m*=1: AIC (Akaike's information criterion) = 28991.97; BIC (Bayesian information criterion) = 29239.14; area under the ROC curve= 0.6095; % correctly classified = 58.08%. CVD: cardiovascular disease; DBP: diastolic blood pressure; IMD: index of multiple deprivation; SBP: systolic blood pressure
