# Supplementary material for: Examining the uptake, retention, and effectiveness of a national online type 2 diabetes self-management intervention in England (Healthy Living): A retrospective cohort study
Source: PLoS One. 2026 Jun 3;21(6):e0348266. doi: 10.1371/journal.pone.0348266 (PMC13232854; doi:10.1371/journal.pone.0348266)
Supplement: S3 Table — Defined as the total number of sessions on the website visited per participant throughout the programme (N = 21,445 HL participants with usage data). (PDF) [file pone.0348266.s003.pdf]

Table S3. Logistic regression model for 'Attendance' using visits frequency measure as binary outcome of session number =1 session (reference group) vs. >1 session. Defined as the total number of sessions on the website visited per participant throughout the programme (N= 21,445 HL participants with usage data)

- Visits frequency = 1 session, N= 12,110 (reference group)
- Visits frequency > 1 session, N= 9,335

Variable	Odds Ratio	Std. Err.	t	P>|t|	[95% Conf. Interval]
**Age**	1.011	0.002	6.63	0.000	1.008 – 1.014
**Sex (**Female**)**	1.096	0.034	2.91	0.004	1.030 – 1.165
**Ethnicity** (Vs. White)					
Asian	0.656	0.036	-7.61	0.000	0.588 – 0.731
Black	0.908	0.066	-1.33	0.185	0.787 – 1.047
Mixed	0.957	0.134	-0.32	0.752	0.728 – 1.258
Other	0.804	0.122	-1.44	0.149	0.597 – 1.081
**IMD quintile (**Vs. IMD Q1 Most deprived**)**					
Q2	1.065	0.049	1.37	0.169	0.973 – 1.166
Q3	1.109	0.051	2.26	0.024	1.014 – 1.213
Q4	1.080	0.049	1.72	0.086	0.989 – 1.180
Q5 (Least deprived)	1.173	0.055	3.41	0.001	1.070 – 1.286
**Smoking status** (vs. Never smoker)					
Current smoker	0.921	0.055	-1.39	0.167	0.819 – 1.035
Ex-smoker	1.027	0.034	0.82	0.410	0.963 – 1.096
Non-smoker (unknown)	0.945	0.098	-0.54	0.586	0.771 – 1.159
**BMI**, kg/m2	0.998	0.002	-0.79	0.431	0.994 – 1.003
**Diabetes duration,** years	0.976	0.003	-9.34	0.000	0.971 – 0.981
**Baseline HbA1c,** mmol/mol	1.002	0.001	2.34	0.020	1.000 – 1.004
**Baseline total cholesterol,** mmol/L	0.997	0.008	-0.37	0.710	0.981 – 1.013
**Baseline SBP,** mmHg	0.999	0.001	-0.89	0.373	0.996 – 1.001
**Baseline DBP,** mmHg	0.997	0.002	-1.64	0.103	0.993 – 1.001
**Baseline serum creatinine,** µmol/L	1.000	0.001	0.07	0.944	0.999 – 1.001
**Ischaemic heart disease (IHD)**	0.932	0.056	-1.17	0.242	0.829 – 1.048
**History of CVD admission**	1.012	0.066	0.19	0.852	0.891 – 1.150
**Learning disability (LD)**	0.826	0.262	-0.60	0.547	0.444 – 1.537
**Severe mental illness (SMI)** (vs. SMI not provided)					
Bipolar disorder	0.854	0.124	-1.09	0.274	0.643 – 1.134
Schizophrenia	0.957	0.203	-0.21	0.836	0.631 – 1.451
Other psychosis	0.821	0.266	-0.61	0.543	0.435 – 1.550
**Baseline antihypertensives**	1.031	0.033	0.96	0.339	0.968 – 1.098
**Baseline insulin**	0.927	0.047	-1.50	0.134	0.839 – 1.024
**Baseline non-insulin antidiabetic agents**	0.729	0.027	-8.59	0.000	0.678 – 0.783
**Baseline statins**	0.860	0.029	-4.47	0.000	0.806 – 0.919
**_cons (Constant)**	0.880	0.197	-0.57	0.568	0.567 – 1.366

In accordance with mandatory data provider Statistical Disclosure Control (SDC) rules (such as, rounding and small number suppression), numbers may not sum to the total.

Model goodness of fit measured on imputation *m*=1: AIC (Akaike's information criterion) = 28918.71; BIC (Bayesian information criterion) = 29165.87; area under the ROC curve= 0.5874; % correctly classified= 58.46%. CVD: cardiovascular disease; DBP: diastolic blood pressure; IMD: index of multiple deprivation; SBP: systolic blood pressure.
