# Supplementary material for: Examining the uptake, retention, and effectiveness of a national online type 2 diabetes self-management intervention in England (Healthy Living): A retrospective cohort study
Source: PLoS One. 2026 Jun 3;21(6):e0348266. doi: 10.1371/journal.pone.0348266 (PMC13232854; doi:10.1371/journal.pone.0348266)
Supplement: S4 Table — (PDF) [file pone.0348266.s004.pdf]

Table S4. Baseline characteristics of the 1:5 matched HL cases-controls cohort study Groups 1 and 3

	NDA Controls (Group 1) N=24,685*	HL account registrants (Group 2) N=6,670	HL account activators (Group 3) N=4,940
**Age** (years), mean (±SD)	58.8 (±13.8)	59.3 (±11.4)	58.6 (±11.3)
**Sex**, N (%)			
Male	10,165 (41%)	2,765 (41%)	2,040 (41%)
Female	14,525 (59%)	3,905 (59%)	2,905 (59%)
**Ethnicity**, N (%)			
Asian	1,775 (7%)	540 (8%)	375 (8%)
Black	640 (3%)	195 (3.0%)	135 (3%)
Mixed	260 (1%)	75 (1%)	50 (1%)
Other	225 (0.9%)	70 (1%)	50 (1%)
White	21,795 (88%)	5,800 (87%)	4,335 (88%)
**Deprivation (IMD)**, N (%)			
IMD Q1 (Most deprived)	4,640 (19%)	1,295 (19.4%)	940 (19.0%)
IMD Q2	4,570 (18%)	1,280 (19.2%)	925 (18.7%)
IMD Q3	5,175 (21%)	1,390 (20.8%)	1,035 (20.9%)
IMD Q4	5,300 (22%)	1,435 (21.5%)	1,055 (21.3%)
IMD Q5 (Least deprived)	5,015 (20%)	1,280 (19.2%)	990 (20.0%)
**Baseline BMI (**kg/ m^2)			
Mean (±SD)	34.1 (±7.8)	34.0 (7.4)	34.2 (±7.6)
Median (IQR)	33.2 (IQR: 28.7, 38.5)	33.0 (28.8, 38.2)	33.2 (IQR: 28.9, 38.6)
**Smoking status**, N (%)			
Current smoker	3,880 (16%)	595 (9%)	430 (9%)
Ex-smoker	8,710 (35%)	2,395 (36%)	1,775 (36%)
Non-smoker (history unknown)	525 (2%)	140 (2%)	100 (2%)
Never smoker	11,580 (47%)	3,540 (53%)	2,640 (54%)
**Diabetes duration,** years			
Mean (±SD)	7.7 (±7.4)	7.1 (7.0)	6.8 (±6.8)
Median (IQR)	6.0 (IQR: 2.0, 12.0)	5.0 (1.0, 11.0)	5.0 (IQR: 1.0, 11.0)
**Referral Route**, N (%)			
Public beta - Hub referral (NWL)	N/A	275 (4.1%)	180 (3.6%)
Self-Referral landing page (Private)	N/A	5,090 (76.3%)	3,715 (75.2%)
Self-Referral landing page (Public)	N/A	1,310 (19.6%)	1,045 (21.1%)
**Baseline HbA1c** mmol/mol, mean (±SD)	62.7 (±19.4)	62.7 (±18.6)	62.71 (±18.5)
**Baseline HbA1c** %, mean (±SD)	7.9 (±1.8)	7.9 (±1.7)	7.89 (±1.7)
**Baseline SBP**, mmHg, mean (±SD)	131.6 (±14.0)	131.7 (±13.3)	131.49 (±13.2)
**Baseline DBP,** mmHg, mean (±SD)	77.7 (±9.4)	78.3 (±9.2)	78.34 (±9.1)
**Baseline total cholesterol,** mmol/L			
Mean (±SD)	4.3 (±1.3)	4.3 (±1.3)	4.3 (±1.3)
Median (IQR)	4.3 (IQR: 3.5, 5.1)	4.2 (3.4, 5.1)	4.2 (IQR: 3.5, 5.2)
**Baseline serum creatinine,** μmol/L, mean (±SD)	76.4 (±33.0)	75.0 (±30.2)	74.1 (±26.7)
**Offered DM education programme**			
Yes	15,965 (65%)	5,240 (79%)	3,895 (79%)
Unspecified	8,725 (35%)	1,430 (21%)	1,045 (21%)
**Attended DM education programme**			
Yes	2,895 (12%)	1,470 (22%)	1,115 (23%)
Unspecified	21,800 (88%)	5,200 (78%)	3,825 (77%)
**Ischaemic heart disease (IHD)**			
Yes	2,735 (11%)	670 (10%)	460 (9%)
Unknown	21,955 (89%)	6,000 (9%)	4,480 (91%)
**History of CVD admission**			

	NDA Controls<br>(Group 1)<br>N=24,685*	HL account registrants<br>(Group 2)<br>N=6,670	HL account activators<br>(Group 3)<br>N=4,940
Yes	275 (1%)	485 (7%)	340 (7%)
Unknown	24,410 (99%)	6,185 (93%)	4,600 (93%)
**Learning disability (LD)**			
Yes	315 (1%)	15 (0.2%)	5 (0.1%)
Unknown	24,375 (99%)	6,655 (99.8%)	4,930 (99.9%)
**Severe mental illness (SMI)**			
Bipolar disorder	290 (1%)	75 (1%)	60 (1%)
Schizophrenia	380 (1.5%)	35 (0.5%)	25 (0.5%)
Other psychosis	90 (0.4%)	15 (0.2%)	10 (0.2%)
SMI Dx not provided	23,935 (97%)	6,555 (98%)	4,850 (98%)
**Baseline medications**, N (%)			
Antihypertensives	15,265 (62%)	4,135 (62%)	3,000 (61%)
Insulin	3,470 (14%)	800 (12%)	580 (12%)
Non-insulin diabetes drugs	17,045 (69%)	5,265 (79%)	3,910 (79%)
Statins	15,570 (63%)	4,400 (66%)	3,180 (64%)
Receiving eight care processes, N (%)			
Yes	8,840 (36%)	14,295 (53%)	2,780 (56%)
No	15,850 (64%)	12,715 (47%)	2,160 (44%)

The table presents imputed data (imputation #1). Based on the controls matched to Group 3.
In accordance with mandatory data provider Statistical Disclosure Control (SDC) rules (such as, rounding and small number suppression), individual categories may not sum to the total, and percentages may not sum to 100%.
BMI: body mass index; CVD: cardiovascular disease; DBP: diastolic blood pressure; HbA1c: glycated haemoglobin; HL: Healthy Living; IHD: ischaemic heart disease; IMD Q: index of multiple deprivation quintile; NDA: National Diabetes audit; SBP: systolic blood pressure; DM: diabetes.

*Based on the controls matched to Group 3.
