# Supplementary material for: Examining the uptake, retention, and effectiveness of a national online type 2 diabetes self-management intervention in England (Healthy Living): A retrospective cohort study
Source: PLoS One. 2026 Jun 3;21(6):e0348266. doi: 10.1371/journal.pone.0348266 (PMC13232854; doi:10.1371/journal.pone.0348266)
Supplement: S5 Table — (PDF) [file pone.0348266.s005.pdf]

Table S5. Baseline characteristics of the 1:5 matched HL cases-controls cohort- groups 1 & 4 and groups 1 and 5

	NDA Controls (Group 1) N=8,290	HL attendees (Group 4) N=1,660	NDA Controls (Group 1) N=1,215	HL completers (Group 5) N=245
**Age, (**years**)**mean (±SD)	58.0 (±13.8)	58.5 (±11.1)	57.7 (±13.8)	58.7 (±10.5)
**Sex**, N (%)				
Male	3,245 (39%)	590 (35%)	410 (33%)	80 (32%)
Female	5,050 (61%)	1,070 (65%)	810 (67%)	165 (68%)
**Ethnicity**, N (%)				
Asian	475 (6%)	75 (5%)	85 (7%)	15 (5%)
Black	185 (2%)	40 (2%)	35 (3%)	5 (2%)
Mixed	75 (1%)	10 (0.6%)	15 (1%)	5 (2%)
Other	75 (1%)	15 (0.7%)	15 (1%)	0
White	7,490 (90%)	1,525 (92%)	1,075 (88%)	230 (93%)
**Deprivation (IMD)**, N (%)				
IMD Q1 (Most deprived)	1,555 (19%)	315 (19%)	230 (19%)	45 (18%)
IMD Q2	1,495 (18%)	285 (17%)	210 (17%)	50 (19%)
IMD Q3	1,710 (21%)	355 (21%)	270 (22%)	50 (20%)
IMD Q4	1,805 (22%)	360 (22%)	270 (22%)	50 (19%)
IMD Q5 (Least deprived)	1,735 (21%)	350 (21%)	250 (20%)	60 (24%)
**Baseline BMI,** (kg/ m^2)				
Mean (±SD)	34.5 (±7.8)	34.3 (±7.6)	34.8 (±7.9)	34.0 (±7.2)
Median (IQR)	33.5 (29.0, 39.0)	33.3 (29.3, 38.5)	33.8 (29.0, 39.5)	33.3 (29.1, 38.1)
**Smoking status**, N (%)				
Current smoker	1,300 (16%)	145 (8%)	185 (15%)	20 (7%)
Ex-smoker	2,910 (35%)	605 (37%)	410 (34%)	95 (39%)
Non-smoker (history unknown)	165 (2%)	30 (2%)	25 (2%)	5 (2%)
Never smoker	3,920 (47%)	885 (53%)	605 (49%)	130 (53%)
**Diabetes duration,** years				
Mean (±SD)	7.5 (±7.2)	6.06 (±6.4)	7.6 (±7.3)	5.6 (±6.6)
Median (IQR)	6.0 (2.0, 11.0)	4.00 (1.0, 10.0)	6.00 (2.0, 11.0)	3.0 (0.0, 9.0)
**Referral Route**				
Public beta - Hub referral (NWL)	N/A	23 (1.4%)	N/A	5 (2%)
Self-Referral landing page (Private)	N/A	1295 (78.1%)	N/A	175 (71.2%)
Self-Referral landing page (Public)	N/A	340 (20.5%)	N/A	70 (28.4%)
**Baseline HbA1c** mmol/mol, mean (±SD)	63.2 (±19.8)	62.7 (±18.5)	62.8 (±19.2)	64.1 (±21.4)
**Baseline HbA1c** %, mean (±SD)	7.9 (±1.8)	7.9 (±1.7)	7.9 (±1.8)	8.02 (±2.0)
**Baseline SBP**, mmHg, mean (±SD)	131.7 (±14.1)	131.6 (±13.3)	131.6 (±13.9)	131.9 (±13.4)
**Baseline DBP,** mmHg, mean (±SD)	77.9 (±9.2)	78.5 (±9.3)	78.3 (±9.3)	78.4 (±9.6)
**Baseline total cholesterol,** mmol/L,				
Mean (±SD)	4.4 (±1.3)	4.4 (±1.3)	4.4 (±1.3)	4.3 (±1.3)
Median (IQR)	4.3 (3.5, 5.2)	4.3 (3.5, 5.3)	4.4 (3.5, 5.2)	4.2 (3.4, 5.2)
**Baseline serum creatinine,** μmol/L, mean (±SD)	75.1 (±30.4)	72.1 (±25.1)	73.3 (±27.0)	68.1 (±22.4)
**Offered DM education programme**				
Yes	5,375 (65%)	1,310 (79%)	790 (65%)	205 (83%)
Unspecified	2,915 (35%)	350 (21%)	430 (35%)	45 (17%)
**Attended DM education programme**				
Yes	1,010 (12%)	395 (24%)	155 (13%)	55 (23%)
Unspecified	7,285 (88%)	1,270 (76%)	1,060 (87%)	190 (77%)
**Comorbidities**, N (%)				
**Ischaemic heart disease (IHD)**				

	NDA Controls (Group 1) N=8,290	HL attendees (Group 4) N=1,660	NDA Controls (Group 1) N=1,215	HL completers (Group 5) N=245
Yes	870 (10%)	140 (8%)	110 (9%)	20 (8%)
Unknown	7,425 (90%)	1,525 (92%)	1,110 (91%)	225 (92%)
**History of CVD admission**				
Yes	95 (1%)	110 (6%)	15 (1%)	15 (6%)
Unknown	8,200 (99%)	1,555 (94%)	1,200 (99%)	230 (94%)
**Learning disability (LD)**				
Yes	120 (1%)	5 (0.3%)	20 (1%)	5 (2%)
Unknown	8,175 (99%)	1,660 (99.9%)	1,200 (99%)	245 (99.6%)
**Severe mental illness (SMI)**				
Bipolar disorder	95 (1%)	15 (1%)	20 (1%)	5 (2%)
Schizophrenia	140 (1.6%)	5 (0.3%)	20 (2%)	0 (0.0%)
Other psychosis	30 (0.3%)	5 (0.3%)	5 (0.4%)	0 (0.0%)
SMI Dx not provided	8,040 (97%)	1,645 (99%)	1,180 (97%)	240 (99%)
**Baseline medications**, N (%)				
Antihypertensives	5,085 (61%)	985 (59%)	730 (60%)	150 (60%)
Insulin	1,165 (14%)	165 (10%)	160 (13%)	35 (14%)
Non-insulin diabetes drugs	5,790 (70%)	1,245 (75%)	860 (71%)	185 (75%)
Statins	5,095 (62%)	1,020 (62%)	725 (60%)	155 (64%)
**Completing eight care processes, N (%)**				
Yes	2,880 (35%)	975 (59%)	420 (34%)	160 (64%)
No	5,410 (65%)	685 (41%)	805 (66%)	90 (36%)

In accordance with mandatory data provider Statistical Disclosure Control (SDC) rules (such as, rounding and small number suppression), individual categories may not sum to the total, and percentages may not sum to 100%.

BMI: body mass index; CVD: cardiovascular disease; DBP: diastolic blood pressure; HbA1c: glycated haemoglobin; HL: Healthy Living; IHD: ischaemic heart disease; IMD Q: index of multiple deprivation quintile; NDA: National Diabetes audit; SBP: systolic blood pressure; DM: diabetes.
