# Supplementary material for: Examining the uptake, retention, and effectiveness of a national online type 2 diabetes self-management intervention in England (Healthy Living): A retrospective cohort study
Source: PLoS One. 2026 Jun 3;21(6):e0348266. doi: 10.1371/journal.pone.0348266 (PMC13232854; doi:10.1371/journal.pone.0348266)
Supplement: S6 Table — (PDF) [file pone.0348266.s006.pdf]

Table S6. Linear regression model output for 1-year HbA1c (mmol/mol) - primary outcome (N= 29,625)

	Coefficient	Std. error	t	P>|t|	95% confidence interval	
**HL activators (vs. NDA Control)**	-1.3	0.220	-5.83	0.000	-1.7	-0.8
**Age**	-0.1	0.009	-8.72	0.000	-0.101	-0.063
**Sex**						
Female	-0.5	0.187	-2.54	0.011	-0.8	-0.1
**Ethnicity** (vs. white)						
Asian	-0.2	0.343	-0.53	0.594	-0.9	0.5
Black	0.04	0.587	0.07	0.944	-1.1	1.2
Mixed	-0.2	0.978	-0.22	0.825	-2.1	1.7
Other	0.4	0.893	0.46	0.644	-1.3	2.2
**IMD** (vs. Q1 Most deprived)						
Q2	-0.7	0.292	-2.37	0.019	-1.3	-0.1
Q3	-0.6	0.297	-2.16	0.032	-1.2	-0.1
Q4	-0.9	0.274	-3.29	0.001	-1.4	-0.4
Q5 (Least deprived)	-0.8	0.330	-2.51	0.015	-1.5	-0.2
**Smoking** (vs. Never smoker)						
Current smoker	0.01	0.296	0.05	0.963	-0.6	0.6
Ex-smoker	-0.2211	0.215	-0.98	0.330	-0.6	0.2
Non-smoker (history unknown)	-0.4	0.732	-0.59	0.560	-1.9	1.0
**BMI** (kg/m^2)	0.04	0.013	3.45	0.001	0.02	0.1
**Diabetes duration** (years)	0.3	0.015	19.51	0.000	0.3	0.3
**HbA1c** (mmol/mol)	0.6	0.007	90.78	0.000	0.6	0.6
**Cholesterol** (mmol/L)	0.4	0.089	4.68	0.000	0.2	0.6
**Systolic BP** (mm Hg)	0.02	0.008	2.09	0.038	0.001	0.03
**Diastolic BP** (mm Hg)	0.02	0.014	1.69	0.099	-0.01	0.1
**Serum creatinine**	-0.01	0.003	-3.14	0.002	-0.02	-0.004
**Ischaemic heart disease (IHD)**						
Yes (vs. unknown)	0.09	0.285	0.31	0.755	-0.5	0.7
**History of CVD admission**						
Yes (vs. unknown)	-0.4	0.626	-0.65	0.518	-1.6	0.8
**Learning disability (LD)**						
Yes (vs. unknown)	0.08	0.915	0.09	0.932	-1.7	1.9
**Severe mental illness (SMI)**						
(vs. Dx not provided)						
Bipolar disorder	-0.7	0.825	-0.88	0.378	-2.3	0.9
Schizophrenia	-2.1	0.840	-2.46	0.015	-3.7	-0.4
Other psychosis	-0.9	1.605	-0.58	0.559	-4.1	2.2
**Baseline medications**						
Antihypertensive drugs	-0.1	0.222	-0.28	0.779	-0.5	0.4
Insulin	1.8	0.304	5.89	0.000	1.2	2.4
Non-insulin diabetes drug	0.9	0.194	4.51	0.000	0.5	1.3
Statins	1.1	0.214	5.34	0.000	0.7	1.6
_cons	18.632	1.480	12.59	0.000	15.681	21.584

BMI: body mass index; CVD: cardiovascular disease; DBP: diastolic blood pressure; HbA1c: glycated haemoglobin; HL: Healthy Living; IHD: ischaemic heart disease; IMD Q: index of multiple deprivation quintile; NDA: National Diabetes audit; SBP: systolic blood pressure; DM: diabetes.
