# Supplementary material for: Examining the uptake, retention, and effectiveness of a national online type 2 diabetes self-management intervention in England (Healthy Living): A retrospective cohort study
Source: PLoS One. 2026 Jun 3;21(6):e0348266. doi: 10.1371/journal.pone.0348266 (PMC13232854; doi:10.1371/journal.pone.0348266)
Supplement: S7 Table — (N = 29,625). (PDF) [file pone.0348266.s007.pdf]

Table S7. Multivariable-adjusted difference (95% CI) in 1-year outcomes in HL account activators (cases) compared with matched NDA controls, excluding covariates with more than 5 or 10 percent imbalances between cases and controls. (N= 29,625)

	Full model with all covariates	Models excluding covariates with ≥10% imbalance*	Models excluding covariates with ≥5% imbalance**
**Linear regression models (beta coefficient, 95% CI)**			
HbA1c (mmol/mol)	-1.3 (-1.7; -0.8)	-1.2 (-1.6; -0.8)	-1.2 (-1.6; -0.7)
Body mass index (BMI), kg/m^2	-0.2 (-0.3; -0.1)	-0.2 (-0.3; -0.1)	-0.2 (-0.3; -0.1)
Systolic blood pressure (SBP), mmHg	-1.2 (-1.6; -0.7)	-1.3 (-1.8; -0.9)	-1.4 (-1.8; -0.9)
Diastolic blood pressure (DBP), mmHg	-0.6 (-0.9; -0.3)	-0.6 (-0.9; -0.3)	-0.6 (-0.9; -0.3)
**Logistic regression models (OR, 95% CI)**			
Insulin use	1.0 (0.8; 1.2)	1.0 (0.8; 1.3)	1.0 (0.8; 1.3)

HbA1c: glycated haemoglobin; HL: Healthy Living; NDA: National Diabetes audit; OR: odds ratio.

Models were adjusted for: age, sex (reference category: male), ethnicity (reference category: White), IMD quintiles (reference category: most deprived), smoking status (reference category: never smoked), BMI, and T2DM duration; baseline ischemic heart disease (reference category: unknown), history of cardiovascular disease admission (reference category: unknown), learning disability (reference category: unknown), and severe mental illness (reference category: diagnosis not provided); baseline prescriptions of antihypertensives, insulin, non-insulin diabetes medications, and statins.

* Variables with ≥10% imbalance: Smoking, cardiovascular disease, foot exam, learning disability, non-insulin diabetes drugs, and severe mental illness.

** Variables with ≥5% imbalance: as above + Statins prescriptions.
