# Supplementary material for: Examining the uptake, retention, and effectiveness of a national online type 2 diabetes self-management intervention in England (Healthy Living): A retrospective cohort study
Source: PLoS One. 2026 Jun 3;21(6):e0348266. doi: 10.1371/journal.pone.0348266 (PMC13232854; doi:10.1371/journal.pone.0348266)
Supplement: S8 Table — (PDF) [file pone.0348266.s008.pdf]

Table S8. Multivariable-adjusted difference (95% CI) in 1-year HbA1c model limited to participants with complete non-missing data Using matched HL activators and NDA controls cohort) (complete case analysis, CCA)

	**Full model (N= 29,625)**	**complete case analysis (CCA) (N=6,620)**
1-year HbA1c outcome (mmol/mol)	-1.3 (-1.7; -0.8)	0.04 (0.7; -0.6)

Models were adjusted for: age, sex (reference category: male), ethnicity (reference category: White), IMD quintiles (reference category: most deprived), smoking status (reference category: never smoked), BMI, and T2DM duration; baseline ischemic heart disease (reference category: unknown), history of cardiovascular disease admission (reference category: unknown), learning disability (reference category: unknown), and severe mental illness (reference category: diagnosis not provided); baseline prescriptions of antihypertensives, insulin, non-insulin diabetes medications, and statins.
