# Supplementary material for: Examining the uptake, retention, and effectiveness of a national online type 2 diabetes self-management intervention in England (Healthy Living): A retrospective cohort study
Source: PLoS One. 2026 Jun 3;21(6):e0348266. doi: 10.1371/journal.pone.0348266 (PMC13232854; doi:10.1371/journal.pone.0348266)
Supplement: S10 Table — (PDF) [file pone.0348266.s010.pdf]

Table S10. Multivariable-adjusted difference (95% CI) in 1-year outcomes in HL account activators, HL attendees and HL completers compared with NDA controls

1-year outcome	HL account activators (Group 3) (N= 29,625) *	HL attendees (Group 4) (N= 9,950) **	HL completers (Group 5) (N= 1,460) ***
**Linear regression models (beta coefficient, 95% CI)**			
HbA1c (mmol/mol)	-1.3 (-1.7; -0.8)	-2.3 (-3.0; -1.6)	-4.0 (-6.1; -1.9)
HbA1c (%)	-0.1 (-0.2; -0.1)	-0.2 (-0.3; -0.1)	-0.4 (-0.6; -0.002)
Body mass index (BMI), kg/m^2	-0.2 (-0.3; -0.1)	-0.4 (-0.5; -0.2)	-0.6 (-1.1; -0.2)
Systolic blood pressure (SBP), mmHg	-1.2 (-1.6; -0.7)	-1.6 (-2.3; -0.8)	-2.2 (-4.4; -0.03)
Diastolic blood pressure (DBP), mmHg	-0.6 (-0.9; -0.3)	-0.6 (-1.1; -0.03)	-0.5 (-1.9; 0.9)
**Logistic regression models (OR, 95% CI)**			
Insulin use	1.0 (0.8; 1.2)	1.4 (1.0; 2.0)	Model did not converge small sample.
Completion of eight care processes	1.6 (1.5; 1.8)	1.9 (1.7; 2.2)	1.9 (1.4; 2.6)

* N= 4,940 HL cases (activators) matched to 24,685 controls (Total =29,625 participants)
**N= 1,660 HL cases (attendees) matched to 8,290 controls (Total =9,950 participants)
*** N= 245 HL cases (completers) matched to 1,215 controls (Total = 1,460 participants)

HbA1c: glycated haemoglobin; HL: Healthy Living; NDA: National Diabetes audit; OR: odds ratio.

Models were adjusted for: age, sex (reference category: male), ethnicity (reference category: White), IMD quintiles (reference category: most deprived), smoking status (reference category: never smoked), BMI, and T2DM duration; baseline ischemic heart disease (reference category: unknown), history of cardiovascular disease admission (reference category: unknown), learning disability (reference category: unknown), and severe mental illness (reference category: diagnosis not provided); baseline prescriptions of antihypertensives, insulin, non-insulin diabetes medications, and statins.
