# Supplementary material for: Examining the uptake, retention, and effectiveness of a national online type 2 diabetes self-management intervention in England (Healthy Living): A retrospective cohort study
Source: PLoS One. 2026 Jun 3;21(6):e0348266. doi: 10.1371/journal.pone.0348266 (PMC13232854; doi:10.1371/journal.pone.0348266)
Supplement: S11 Table — (PDF) [file pone.0348266.s011.pdf]

Table S11. Multivariable-adjusted difference (95% CI) in 1-year outcomes in matched HL cases and NDA controls across different HL development phases

Regression models based on participants in different HL development phases

1-year outcome	**Matched cohort including all HL account activators (Group 3) (N= 29,625) ***	**Matched cohort including HL participants part of the development/testing phase (N= 17,335) ****	**Matched cohort including HL participants part of the finalised version (N= 12,290) *****
**Linear regression models (beta coefficients, 95% CI)**			
HbA1c (mmol/mol)	-1.3 (-1.7; -0.8)	-1.3 (-1.8; -0.7)	-1.3 (-1.9; -0.6)
HbA1c (%)	-0.1 (-0.2; -0.1)	-0.1 (-0.2; -0.1)	-0.1 (-0.2; -0.1)
Body mass index (BMI), kg/m^2	-0.2 (-0.3; -0.1)	-0.2 (-0.4; -0.1)	-0.2 (-0.4; -0.02)
Systolic blood pressure (SBP), mmHg	-1.2 (-1.6; -0.7)	-1.1 (-1.6; -0.5)	-1.3 (-2.1; -0.6)
Diastolic blood pressure (DBP), mmHg	-0.6 (-0.9; -0.3)	-0.5 (-0.9; -0.1)	-0.7 (-1.3; -0.1)
**Logistic regression models (OR, 95% CI)**			
Insulin use	1.0 (0.8; 1.2)	1.1 (0.8; 1.4)	0.9 (0.6; 1.2)
Completion of eight care processes	1.6 (1.5; 1.8)	1.5 (1.4; 1.6)	1.8 (1.7; 2.1)

* N= 4,940 HL cases (activators) matched to 24,685 controls (Total =29,625 participants)
**N= 2,890 HL cases matched (activators) to 14,445 controls (Total =17,335 participants).
***N= 2,050 HL cases matched (activators) to 10,240 controls (Total =12,290 participants).

HbA1c: glycated haemoglobin; HL: Healthy Living; NDA: National Diabetes audit; OR: odds ratio.

Models were adjusted for: age, sex (reference category: male), ethnicity (reference category: White), IMD quintiles (reference category: most deprived), smoking status (reference category: never smoked), BMI, and T2DM duration; baseline ischemic heart disease (reference category: unknown), history of cardiovascular disease admission (reference category: unknown), learning disability (reference category: unknown), and severe mental illness (reference category: diagnosis not provided); baseline prescriptions of antihypertensives, insulin, non-insulin diabetes medications, and statins.
