# Supplementary material for: Examining the uptake, retention, and effectiveness of a national online type 2 diabetes self-management intervention in England (Healthy Living): A retrospective cohort study
Source: PLoS One. 2026 Jun 3;21(6):e0348266. doi: 10.1371/journal.pone.0348266 (PMC13232854; doi:10.1371/journal.pone.0348266)
Supplement: S12 Table — (PDF) [file pone.0348266.s012.pdf]

Table S12. Comparison of results of selected outcomes with results from the HeLP-Diabetes trial

**HED-LINE study**						
	**HL activators**		**Control**		**HL vs. Control**	
1-year outcome	Baseline	Change from baseline to 1 year	Baseline	Change from baseline to 1 year	Mean difference (95% CI)	p Value
HbA1c, mmol/mol	62.71	-2.4	62.71	-1.1	-1.3 (-1.8; -0.9)	<0.0001
HbA1c, %	7.89	-0.2	7.89	-0.1	-0.1 (-0.2; -0.1)	<0.0001
Body mass index (BMI), kg/m^2	34.18	-0.4	34.14	-0.2	-0.2 (-0.3; -0.1)	<0.0001
Systolic blood pressure (SBP), mmHg	131.49	+0.1	131.64	+1.4	-1.2 (-1.7; -0.8)	<0.0001
Diastolic blood pressure (DBP), mmHg	78.34	-0.2	77.74	+0.8	-0.9 (-1.2; -0.6)	<0.0001
Insulin use (no baseline insulin)*	11.7%	-9.6%	14%	-11.9%	OR: 1.0 (0.8; 1.2)	0.919
Completion of eight care processes*	56.3%	+2.5%	35.8%	+8.9%	OR: 1.6 (1.5; 1.8)	<0.0001
**HeLP-Diabetes trial**						
	**HeLP-Diabetes**		**Control**		**HeLP-Diabetes vs control**	
1-year outcome	Baseline	Change from baseline to 1 year	Baseline	Change from baseline to 1 year	Mean difference (95% CI)	p Value
HbA1c, mmol/mol	56.3	−0.8	56.8	+1.8	−2.6 (−4.8; -0.5)	0.014
HbA1c, %	7.3	−0.1	7.3	+0.2	−0.2 (−0.4; -0.1)	0.014
Body mass index (BMI), kg/m^2	30.1	+0.1	30.0	−0.04	0.2 (−0.3; 0.6)	0.498
Systolic blood pressure (SBP), mmHg	134.7	−4.2	134.9	−0.5	−3.8 (−6.6; -0.9)	0.010
Diastolic blood pressure (DBP), mmHg	77.8	−2.5	77.1	−1.9	−0.6 (−2.4; 1.2)	0.519
Completion of 9 care processes*	65%	−5.1%	61%	+3.4%	OR: 0.8 (0.5; 1.4)	0.379
