# Supplementary material for: Examining the uptake, retention, and effectiveness of a national online type 2 diabetes self-management intervention in England (Healthy Living): A retrospective cohort study
Source: PLoS One. 2026 Jun 3;21(6):e0348266. doi: 10.1371/journal.pone.0348266 (PMC13232854; doi:10.1371/journal.pone.0348266)
Supplement: Attachment — Submitted filename: Response to Reviewers.pdf [file pone.0348266.s015.pdf]

Dear Editor,

Re. PONE-D-25-33501 - Examining the uptake, retention, and effectiveness of a national online type 2 diabetes self-management intervention in England (Healthy Living): a retrospective cohort study

Thank you for the opportunity to submit a revision of our manuscript.

Below, we provide a point-by-point response to the received comments. To facilitate the review process, all changes are highlighted in the revised manuscript and numbered by the Reviewer and comment numbers. For example, "Response to Reviewer 1, comment #1".

We hope to have satisfactorily responded to the comments, which have strengthened our manuscript.

Looking forward to receiving your decision.

Yours sincerely,

Corresponding Author, on behalf of the authors

Academic Editor

Journal Requirements:

When submitting your revision, we need you to address these additional requirements.

I. **Please ensure that your manuscript meets PLOS ONE's style requirements, including those for file naming. The PLOS ONE style templates can be found at**

https://journals.plos.org/plosone/s/file?id=wjVg/PLOSOne_formatting_sample_main_body.pdf [journals.plos.org] and

https://journals.plos.org/plosone/s/file?id=ba62/PLOSOne_formatting_sample_title_authors_affiliations.pdf [track.editorialmanager.com]

Authors response

We reviewed the manuscript and confirm that it meets the PLOS ONE's style requirements.

II. **Thank you for stating the following in the Competing Interests section:**

"MKR reports receiving consulting fees from Eli Lilly and modest GSK stock ownership both unrelated to this work. He has also led research at the University of Manchester with Innovate UK and NIHR funding to independently evaluate My Diabetes My Way, a digital

intervention supporting the management of diabetes. Other authors declare no conflicts of interest."

We note that one or more of the authors are employed by a commercial company: GSK

1. **Please provide an amended Funding Statement declaring this commercial affiliation, as well as a statement regarding the Role of Funders in your study. If the funding organization did not play a role in the study design, data collection and analysis, decision to publish, or preparation of the manuscript and only provided financial support in the form of authors' salaries and/or research materials, please review your statements relating to the author contributions, and ensure you have specifically and accurately indicated the role(s) that these authors had in your study. You can update author roles in the Author Contributions section of the online submission form.**

Please also include the following statement within your amended Funding Statement.

"The funder provided support in the form of salaries for authors [insert relevant initials], but did not have any additional role in the study design, data collection and analysis, decision to publish, or preparation of the manuscript. The specific roles of these authors are articulated in the 'author contributions' section."

If your commercial affiliation did play a role in your study, please state and explain this role within your updated Funding Statement.

Authors response

We confirm that the commercial affiliation did not play any role in this study. The Funding Statement has been revised to declare this commercial affiliation and the proposed statement has been also included. The amended Funding Statement now reads as follows in the manuscript:

"This paper reports independent research funded by the National Institute for Health and Care Research (Policy Research Programme, HED-LINE, NIHR200933). The views expressed in this publication are those of the authors and not necessarily those of the National Institute for Health and Care Research or the Department of Health and Social Care. The funder did not have any role in the study design, data collection and analysis, decision to publish, or preparation of the manuscript. Eli Lilly provided support in the form of consulting fees for MKR, unrelated to this work, and did not have any role in the study design, data collection and analysis, decision to publish, or preparation of the manuscript. The specific role of MKR is articulated in the 'author contributions' section. EK is part-funded by the NIHR HeatlhTech Research Centre in Emergency and Acute Care (NIHR205301) and the Manchester British Heart Foundation (BHF) Centre for Research Excellence (RE/24/130017)."

2. **Please also provide an updated Competing Interests Statement declaring this commercial affiliation along with any other relevant declarations relating to**

employment, consultancy, patents, products in development, or marketed products, etc.

Within your Competing Interests Statement, please confirm that this commercial affiliation does not alter your adherence to all PLOS ONE policies on sharing data and materials by including the following statement: "This does not alter our adherence to PLOS ONE policies on sharing data and materials." (as detailed online in our guide for authors http://journals.plos.org/plosone/s/competing-interests [journals.plos.org]) . If this adherence statement is not accurate and there are restrictions on sharing of data and/or materials, please state these. Please note that we cannot proceed with consideration of your article until this information has been declared.

Please include both an updated Funding Statement and Competing Interests Statement in your cover letter. We will change the online submission form on your behalf.

Authors response

Both updated Funding Statement and Competing Interests Statement has been included in the cover letter. The proposed statement has been added to the Competing Interests Statement, and it now reads as:

"MKR reports receiving consulting fees from Eli Lilly and modest GSK stock ownership both unrelated to this work. He has also led research at the University of Manchester with Innovate UK and NIHR funding to independently evaluate My Diabetes My Way, a digital intervention supporting the management of diabetes. This does not alter our adherence to PLOS ONE policies on sharing data. Other authors declare no conflicts of interest."

3. Thank you for stating the following financial disclosure:

"This paper reports independent research funded by the National Institute for Health and Care Research (Policy Research Programme, HED-LINE, NIHR200933)."

Please state what role the funders took in the study. If the funders had no role, please state: "The funders had no role in study design, data collection and analysis, decision to publish, or preparation of the manuscript."

If this statement is not correct you must amend it as needed.

Please include this amended Role of Funder statement in your cover letter; we will change the online submission form on your behalf.

Authors response

The proposed statement has been added to the Funding Statement, added to the cover letter and it now reads as:

"This paper reports independent research funded by the National Institute for Health and Care Research (Policy Research Programme, HED-LINE, NIHR200933). The views expressed in this publication are those of the authors and not necessarily those of the National Institute for Health and Care Research or the Department of Health and Social Care. The funder did not have any role in the study design, data collection and analysis, decision to publish, or preparation of the manuscript. Eli Lilly provided support in the form of consulting fees for MKR, unrelated to this work, and did not have any role in the study design, data collection and analysis, decision to publish, or preparation of the manuscript. The specific role of MKR is articulated in the 'author contributions' section. EK is part-funded by the NIHR HeatlhTech Research Centre in Emergency and Acute Care (NIHR205301) and the Manchester British Heart Foundation (BHF) Centre for Research Excellence (RE/24/130017)."

4. **We noted in your submission details that a portion of your manuscript may have been presented or published elsewhere. "Yes: 2 text-only abstracts briefly outlining the results were submitted to academic conferences" Please clarify whether this [conference proceeding or publication] was peer-reviewed and formally published. If this work was previously peer-reviewed and published, in the cover letter please provide the reason that this work does not constitute dual publication and should be included in the current manuscript.**

Authors response

Thank you for this comment. We have explained how this work does not constitute dual publication in the cover letter. Both abstracts were peer-reviewed by internal conference committees, but only one abstract was published in *Diabetic Medicine* supplementary issue (Diabetes UK Conference, 2025). Each abstract contains a succinct text-only summary, without detailed methodology, full results, figures/tables. The current manuscript submitted to PLOS ONE includes substantial new content, including full methods, analyses, results, and conclusions, which have never been published elsewhere. No copyright was transferred as part of the conference abstract publication. For transparency, we have fully disclosed the submission of abstracts, and we confirm that this manuscript submission does not constitute duplicate or prior publication under PLOS ONE's policies or ICMJE Recommendations.

5. **Please note that your Data Availability Statement is currently missing the DOI/accession number of each dataset OR a direct link to access each database. If your manuscript is accepted for publication, you will be asked to provide these details on a very short timeline. We therefore suggest that you provide this information now, though we will not hold up the peer review process if you are unable.**

Authors response

The data underlying the results presented in the study are only available through the data provider ‘NHS England’ and authors were not provided with and DOI/accession number. All interested researchers can request to access the data through NHS England directly and authors are not permitted to share the data publicly (even as minimal underlying dataset) or make it available as per the data use agreement with NHS England. The authors did not have any special access privileges to this data.

6. **Please include your full ethics statement in the ‘Methods’ section of your manuscript file. In your statement, please include the full name of the IRB or ethics committee who approved or waived your study, as well as whether or not you obtained informed written or verbal consent. If consent was waived for your study, please include this information in your statement as well.**

Authors response

The following ethics statement is now included in the Methods section of the manuscript as follows: “This study was reviewed and approved by the Yorkshire & The Humber - Leeds West Research Ethics Committee on 18 August 2020 (reference number: 20/YH/0250). The research team undertook secondary analysis of pseudonymised datasets and were not responsible for data collection or obtaining informed consent.”

7. **Please include captions for your Supporting Information files at the end of your manuscript, and update any in-text citations to match accordingly. Please see our Supporting Information guidelines for more information: http://journals.plos.org/plosone/s/supporting-information [journals.plos.org].**

Authors response

Captions for the Supporting Information files are now added at the end of the manuscript, and in-text citations have also been reviewed and updated.

8. **If the reviewer comments include a recommendation to cite specific previously published works, please review and evaluate these publications to determine whether they are relevant and should be cited. There is no requirement to cite these works unless the editor has indicated otherwise.**

Authors response

The reviewers' comments did not recommend including additional references; hence no new citations were added to the manuscript.

Reviewer #1

Abstract (results):

1) **Can the authors indicate which values are crude ORs and the 95%CIs through the results for consistency?**

Authors response

Thank you for this comment. All reported ORs are estimated using multivariable logistic regression models i.e. adjusted ORs. We did not feel univariable associations would add any value to the paper, and any such estimates were not directly relevant to our research question. We have now corrected this in the abstract to indicate that all reported ORS are adjusted ORs.

Changes to the paper

- Abstract: Removed any mention of 'aOR' from the abstract and all are now reported as 'OR' for consistency.
- Methods section (Line 170): the relevant text now reads as "Using multivariable logistic regression models, we estimated adjusted odds ratios (OR) and 95% confidence intervals (95% CI)…"

Main manuscript:

2) **Line 143: Delete fullstop (.)**

Authors response

The full stop has been deleted.

Changes to the paper

The text now reads as: "2.4.1. Uptake and retention of Healthy Living"

3) **Line 186: Do you mean values or variables? If variables, can you explain this was done beyond the stated STATA command used?**

Authors response

We thank the reviewer for this comment. We meant values of baseline variables and have revised the text for clarity.

Changes to the paper

The text now reads as (Line 195): "Multiple imputation using the *mi suite* of commands in Stata was used to impute missing values of baseline variables and outcomes (10 imputations)."

4) Line 221: For consistency, can all 95%CIs be presented in this style?

Authors response

All 95% CIs have been revised and presented as per the suggested style throughout the paper.

5) Line 225: From which table comes this results?

Authors response and Changes to the paper

We would clarify to the reviewer that the reported results of the retention analysis are presented in Figure S1 which is now cited earlier in the text for clarity (Line 253).

6) Line 232: From which table or figure comes this results, i.e. line 232-240?

Authors response

Thank you for this comment and we apologise for the omission.

Changes to the paper

In response to this comment, the full tables have now been added to the supplementary file as Tables S2 and S3 and cited in the main manuscript as follows (Line 264-267):

"We compared longer attendees, who attended for more than the median time (N=10,710), and shorter attendees, who attended for the median or less (N=10,730). Longer attendees were, on average less likely to be male or to be of Asian or Black ethnicity, less likely to be prescribed insulin or non-insulin diabetes medication or statins, and their time since T2DM diagnosis was lower than shorter attendees (S2 Table). People who attended multiple sessions, were on average older, less likely to be male or to be of Asian ethnicity, to be from the most deprived areas, prescribed non-insulin diabetes medication or statins, and their time since T2DM diagnosis was shorter when compared with those who attended one session (S3 Table)."

7) Line 233: Please add the IQR.

Authors response and Changes to the paper

Thank you for this comment. The IQR has now been added to the manuscript and reads as (Line 260):

"….the median time spent on Healthy Living website was 6.9 mins (IQR 0.4 - 26.3)."

All the tables need to be checked for:

8) **Most percentages are not up to 100% or more than 100%. Please check the whole table**

Authors response

We would like to highlight that we have been directed to apply the data provider's Statistical Disclosure Control rules to all reported figures which involved: 'Small Number Suppression' and 'Rounding' figures to the nearest five to prevent identification. As a result, it is standard for totals and percentages to not sum exactly, and we confirm that it does not indicate an error in the analysis or reporting.

Changes to the paper

We have added the following text in the Methods section and as a footnote to all tables to indicate the reason of any apparent total/percentage discrepancies, and it reads as follows:

"In accordance with mandatory data provider Statistical Disclosure Control (SDC) rules (such as, rounding and small number suppression), individual categories may not sum to the total, and percentages may not sum to 100%."

9) **Some figures that needed percentages were not added (e.g. Table S1, Ethnicity - Mixed, and Other). These apply to most percentages.**

Authors response

As above, this is due to rounding and Small Number Suppression as part of the applied Statistical Disclosure Control rules.

Changes to the paper

We have reviewed figures in all tables and added percentages to supressed small numbers in line with the SDC rules.

Reviewer #2

In this paper, the authors reported effectiveness of online intervention in England (Healthy Living; HL), as compared to the matched-controls (NDA). The online intervention resulted in modest improvements in several metabolic parameters after 1 year. The results are almost clearly presented, and the analyses seemed to be well performed. The reviewer's comments are as follows.

1) **The contents of Healthy Living are not clearly described. Some supplementary information would be helpful.**

Authors response

We thank the reviewer for their positive note on our paper and for this comment which we have addressed as outlined below.

Changes to the paper

The following text describing the contents of Healthy Living has been added to the Introduction section of the paper (Line 105-112):

"The Healthy Living website is composed of 895 web pages containing videos, articles, tools, and self-assessment quizzes.[27] The content is broadly divided into three main sections: 1) the 'Learn journey' section: a structured curriculum comprised of different educational modules, 2) the 'Find answers' section: unstructured content section dedicated to different type 2 diabetes-related topics, and 3) the 'Tools' section: includes a range of *Goals* and *Tracker* tools based on the HeLP-Diabetes website where service users can set goals and self-monitor their health."

2) **Please add the description regarding ethical approval by the institution, in the main text.**

Authors response

We thank the reviewer for this comment which we have addressed as outlined below.

Changes to the paper

The following ethics statement is now included in the Methods section of the manuscript (Line 146-150):

"2.3. Ethics statement

This study was reviewed and approved by the Yorkshire & The Humber - Leeds West Research Ethics Committee on 18 August 2020 (reference number: 20/YH/0250). The research team

undertook secondary analysis of pseudonymised datasets and were not responsible for data collection or obtaining informed consent."

3) **The participants in HL program and the matched-controls (NDA) are essentially different. The participants in HL program were younger with shorter duration of diabetes and with lower past history of ischemic heart disease (Table S1). The participants in HL program might have more positive attitude to improve their glucose control. Also, there are no consideration on treatment intensification during the period. Therefore, much more caveats should be added on the improvements in metabolic parameters.**

Authors response

We would like to highlight to the reviewer that the characteristics of the matched HL account activators and NDA controls are presented in S4 Table (not S1 Table) which shows overall balanced parameters between both groups, including age (58.8 vs. 58.6), diabetes duration (7.7y vs. 6.8y) and prevalent IHD (11% vs. 9%). While S1 Table presents all NDA controls (N=3,707,670) who were eligible for inclusion in the study and in the 'uptake and retention' analysis, but only N=24,685 of those were eligible as matched controls were eligible for inclusion in the 'effectiveness' analysis.

We agree with the reviewer that people who choose to participate in the HL program may have a more positive attitude towards improving their glucose control. However, this is not something that can be quantified easily, but we can declare and discuss as a limitation.

No data was available on medication intensification, which acts as a caveat to the reported findings, and it has been now added to the limitations section as below.

Changes to the paper

The following text has been added to the limitations section in response to this comment (Line 387 -390):

"Fifth, while we addressed confounding by controlling for measured characteristics, there may be unmeasured variations between patients which account for the differences in outcomes between groups. Sixth, no data was available to assess the impact of medication intensification on the observed effectiveness findings."

4) **The improvement in HbA1c levels may be smaller than that anticipated. Some more discussion should be made regarding this observation.**

Authors response

Thank you for this comment. The reviewer is likely referring to comparing the observed improvement to the clinically significant reduction in HbA1c. We agree that the mean HbA1c

reduction observed in our study (by 1.3 mmol/mol) is smaller than the 3-5 mmol/mol change generally regarded as clinically meaningful at the individual level. However, even modest reductions in HbA1c levels can result in important population-level benefits due to the evident association between HbA1c levels and development of microvascular and macrovascular complications.

In addition, we have indicated in the manuscript that the study had a few limitations which collectively necessitate cautious interpretation of some of the reported findings, including the primary study outcome: 1-year HbA1c.

Changes to the paper

In the manuscript we wrote, "*An HbA1c reduction of 3-5 mmol/mol [33, 34] is considered to be clinically significant for reducing the risk of diabetes-related complications at the individual patient level.[33] While the mean difference of 1.3 mmol/mol observed in our study is lower, this difference is still likely to provide modest benefits at population level for reducing the risk of developing long-term diabetes-related microvascular and macrovascular complications*."

Since the study was not designed to assess mechanisms of change, it is uncertain why the mean difference was 1.3 mmol/mol and whether this would be maintained over time. However, we have expanded on possible factors in the revised text. If needed, the reviewer and/or editorial team may wish to provide guidance on how this discussion could be further extended.

In response to this comment, the text now reads as (Line 352-359):

"An HbA1c reduction of 3-5 mmol/mol [33, 34] is considered to be clinically significant for reducing the risk of diabetes-related complications at the individual patient level.[33] Although the improvement in HbA1c in our study (1.3 mmol/mol) is smaller, such modest effects are common in real-world evaluations where patient characteristics, varied engagement and attitudes towards glucose control, and routine practice conditions may affect the magnitude of observed change compared to controlled trials. Nonetheless, this small average reduction is still likely to provide modest benefits at population level for reducing the risk of developing long-term diabetes-related microvascular and macrovascular complications."

5) **Results, Figure 1. Were the participants who attended the program (n=8375) included those with HL account (n=21820)? If so, revise Figure 1 to avoid confusion.**

Authors response and Changes to the paper

Thank you for this comment. That is correct, participants who attended the programme are a sub-group of participants who activated their HL account. Figure 1 has been revised accordingly for clarity.
